# From QTL to candidate genes: a data-driven approach to unravel the genetic architecture of yellow rust resistance in central European wheat

**DOI:** 10.1007/s00122-026-05312-8

**Published:** 2026-07-30

**Authors:** Jiaojiao Wang, Renate H. Schmidt, Guoliang Li, Albrecht Serfling, Jochen C. Reif, Yong Jiang

**Affiliations:** 1 Leibniz Institute of Plant Genetics and Crop Plant Research (IPK) Gatersleben, Seeland, Germany; 2 Julius Kühn Institute (Federal Research Centre for Cultivated Plants), Quedlinburg, Germany

## Abstract

***Key message A data mining strategy capitalizing on high-resolution SNP information, sequence variant annotation and QTL information from three populations facilitated an efficient nomination of candidate genes for YR resistance, supported by Yr27 and functional annotations.***

**Abstract** Genome-wide association studies (GWAS) have become routine in many crops, but the prioritization of candidate genes remains challenging. Here, we developed a new approach to identify environment-specific quantitative trait loci (QTL) using GWAS and analyzed 5,840 wheat genotypes distributed over three experimental populations, including 5,243 single-cross hybrids and 597 elite lines. Resequencing the parental genotypes identified over 640,000 single-nucleotide polymorphisms (SNPs) after filtering. The hybrid panels were tested in 19 environments for susceptibility to *Puccinia striiformis* f. sp. *tritici* (*Pst*), a pathogen responsible for severe yellow rust (YR) epidemics especially after 2012. QTL patterns in diverse environments showed substantial differences, reflecting spatial and temporal dynamics. Combining high-resolution SNP information, sequence variant annotation and QTL information from different populations obtained via a novel GWAS approach enabled the efficient nomination of candidate genes for YR resistance loci of particular relevance for Central European wheat. The power of the developed strategy for mining data from different experimental populations was demonstrated as the validated resistance gene *Yr27* was identified as sole candidate gene for one QTL region.

**Supplementary Information:**

The online version contains supplementary material available at 10.1007/s00122-026-05312-8.

## Introduction

The ongoing race between plants developing new resistance genes and pathogens evolving to overcome these defenses is a central challenge in agriculture. Yellow rust (YR), caused by *Puccinia striiformis* f. sp. *tritici* (*Pst*), is a wheat disease that has triggered several epidemics in Central Europe over the past decade, beginning with the emergence of the *Pst* Warrior races (Hovmøller et al. 2016). Gaining a deeper understanding of the genetic architecture and underlying mechanisms is crucial toward developing genotypes conferring durable resistance (Dracatos et al. 2023).

Genome-wide association studies (GWAS) are uniquely suited for dissecting quantitative trait loci (QTL) that contribute to phenotypic variation in traits (Uffelmann et al. 2021). In case of YR, several hundred QTL have been reported, not all of which are unique (Cheng et al. 2024; Tong et al. 2024; Wu et al. 2025). So far, twelve resistance (*R*) genes that can reduce susceptibility to YR have been cloned and characterized (Ayala et al. 2024; Sharma et al. 2024). Among these, *Yr5x*, *Yr6*/*Pm5*, (Wu et al. 2025), *Yr5/YrSP*, *Yr7* (Marchal et al. 2018), *Yr10* (Liu et al. 2014), *Lr13/Yr27* (Hewitt et al. 2021; Yan et al. 2021; Athiyannan et al. 2022), *Yr28/YrAS2388* (Zhang et al. 2019), *YrU1* (Wang et al. 2020), *Yr87/Lr85* (Sharma et al. 2024) and *Yr84/YrTD121* (Klymiuk et al. 2025; Hu et al. 2025) encode nucleotide-binding site (NBS) and leucine-rich repeat (LRR) proteins (NLRs) with varying N-terminal or C-terminal domains; and the remaining genes, *Yr15* (Klymiuk et al. 2018), *Yr36* (Fu et al. 2009), *Lr34*/*Yr18* (Krattinger et al. 2009), *Lr67*/*Yr46* (Moore et al. 2015) and *YrKB* (Wu et al. 2025) code for proteins of different structural classes. Although *NLR* gene-mediated broad-spectrum all-stage resistance has been observed (Sharma et al. 2024), most *NLR* genes are effective against a subset of *Pst* isolates and conform to the classical gene-for-gene model. According to this model, a specific dominant *R* gene encoding a receptor interacts with a matching dominant effector gene in a particular race of a pathogen; thus, new virulent races of a pathogen may overcome this class of *R* genes (Jones et al. 2024). In contrast, the so-called adult plant resistance (*APR*) genes act quantitatively and hence confer only partial resistance at the adult plant stages. *APR* genes include, for example, *Yr36, Lr34*/*Yr18* and *Lr67*/*Yr46* and as they lack recognition triggered immunity motifs typical for *NLR* genes, they are expected to be more durable.

A *Yr* gene catalog, based on pedigree and marker data, as well as virulence/avirulence studies (McIntosh 1995), was expanded using resistance gene enrichment sequencing (RenSeq, Jupe et al. 2013) to compile a resistance gene atlas for European winter wheat (Kale et al. 2022). Owing to the targeted sequencing of *NLR-*related genes, other classes of resistance genes are not included in this resource. To achieve a representative coverage of all different classes of *Yr* genes requires therefore resequencing rather than RenSeq of association panels. Moreover, the resistance gene atlas focused on average performance across environments (Kale et al. 2022). However, a large study involving the German variety registration trials between 1983 and 2019 showed that YR disease occurred on only 29% of the trials, whereas for other diseases much higher values were reported (Laidig et al. 2021). Similarly, variation in marker–trait associations (MTAs) was reported when a multi-parent advanced generation intercross (MAGIC) population was studied for adult plant resistance to YR in field trials in Germany (Rollar et al. 2021). This emphasizes that studying MTAs underlying YR resistance in environments representing a wide temporal and spatial diversity is key to understanding the efficacy of resistance genes. To address this, models for detecting QTL-by-environment (QTL × E) interactions have been gradually developed (Malosetti et al. 2013; Moore et al. 2019; Kerin and Marchini 2020; Eltaher et al. 2021; Branchereau et al. 2023; Lopez-Cruz et al. 2023; Raman et al. 2023; Zhong et al. 2023; Liu et al. 2024), but only a limited number have specifically addressed dominance effects (Li et al. 2022). Incorporating dominance effects into QTL × E studies of disease resistance is advisable, as race-specific resistance genes often exhibit dominant inheritance.

In this study, we analyzed the susceptibility to YR in Central European environments for three different resequenced association panels, which together represent more than 5,000 wheat hybrids. We employed a GWAS approach specifically tailored to explore QTL × E interactions, enabling the identification of QTL that are effective across multiple environments and specific to individual environments, while considering both additive and dominance effects. QTL identified in several environments explained on average a larger proportion of phenotypic variation (PVE) compared to QTL specific for a particular environment. For four loci of particular value for breeding wheat in Central Europe, several thousand significant MTAs were observed, but by applying strict criteria for candidate gene nomination which capitalized on detailed QTL information for different hybrid association panels it was possible to pinpoint one to two genes each for three of these QTL.

## Material and methods

### Plant materials and field trials

In this study, 597 elite winter bread wheat lines and their 5,243 single-cross hybrids were phenotyped for YR resistance in three different experimental series (Exp, Supplemental Table S1). Details of the plant materials have been published previously (Zhao et al. 2021). Briefly, single-cross hybrids were generated by crossing elite wheat lines according to partial factorial designs.

The lines and hybrids were evaluated at five (Exp I) to seven environments (Exp II and Exp III; Supplemental Table S2) following an alpha lattice design with environments as replications (i.e., the trial was unreplicated within each environment). Environments were defined as location × year combinations. In total, there were 11 locations in Germany where the experiments were conducted. Field trials were evaluated between 2012 and 2019, with each experimental series spanning two years. The different Exp were linked by up to 25 common genotypes (Supplemental Table S3).

The severity of YR was assessed in all locations using a standardized experimental procedure following the guidelines of the German Federal Plant Variety Office (Bundessortenamt 2000). Inoculation was done using a mix of strains, as described in detail elsewhere (Longin et al. 2013; Gowda et al. 2014). Data were recorded using a rating scale ranging from 1 (fully resistant) to 9 (fully susceptible).

In addition, the Australian spring wheat cultivar ‘Avocet,’ which is highly susceptible to YR, and 21 genotypes carrying different resistance genes were evaluated in Quedlinburg, Germany, from 2015 to 2024 (see Supplemental Methods for details).

### Phenotypic data analyses

Since there were no replicates within each environment, the genomic repeatability was used as a quality control of the phenotypic data. It was estimated using the genomic best linear unbiased prediction (GBLUP) model (VanRaden 2008). The genomic repeatability was estimated as ${h}_{g}^{2}={\widehat{\sigma }}_{g}^{2}/({\widehat{\sigma }}_{g}^{2}+{\widehat{\sigma }}_{e}^{2})$, where ${\widehat{\sigma }}_{g}^{2}$ and ${\widehat{\sigma }}_{e}^{2}$ are the estimates of the genotypic and residual variance.

To estimate the broad-sense heritability, the following linear mixed model (LMM) was implemented for each experimental series:

1 $$\begin{array}{*{20}c} {y_{ij} = \mu + L_{j} + \varepsilon_{i} g_{i}^{p} + \left( {1 - \varepsilon_{i} } \right)g_{i}^{h} + e_{ij} ,} \\ \end{array}$$

where ${y}_{ij}$ represents the observed score of YR resistance for the *i*th genotype in the *j*th environment, μ corresponds to the general mean, ${L}_{j}\sim N\left(0,{\sigma}_{L}^{2}\right)$ denotes the *j*th environment effect, and ${\varepsilon}_{i}$ took the value 1 if the *i*th genotype is a parental line and 0 if it is a hybrid progeny; if the *i*th genotype is a line, its genotype effect is modeled by ${g}_{i}^{p}\sim N\left(0,{\sigma}_{gp}^{2}\right)$, and if it is a hybrid, its genotype effect is ${g}_{i}^{h}\sim N\left(0,{\sigma}_{gh}^{2}\right)$; ${e}_{ij}\sim N\left(0,{\sigma}_{e}^{2}\right)$ is the residual term. The LMM was fitted by using ASReml-R 4.2 (Gilmour et al. 1995).

The broad-sense heritability for the parental lines (${H}_{\mathrm{p}}^{2}$) and for the hybrids (${H}_{\mathrm{h}}^{2}$) was estimated separately as follows:

2 $$H_{{\mathrm{p}}}^{2} = \sigma_{gp}^{2} {/}\left( {\sigma_{gp}^{2} + \frac{{\sigma_{e}^{2} }}{{n_{Env} }}} \right), H_{{\mathrm{h}}}^{2} = \sigma_{gh}^{2} {/}\left( {\sigma_{gh}^{2} + \frac{{\sigma_{e}^{2} }}{{n_{{{\mathrm{Env}}}} }}} \right),$$

where ${n}_{\mathrm{E}\mathrm{n}\mathrm{v}}$ is the number of environments.

The best linear unbiased estimates (BLUEs) for all genotypes were obtained by fitting model (1) again, but the genotypic effects ${g}_{i}^{p}$ and ${g}_{i}^{h}$ were treated as fixed effects.

The phenotypic correlations between all pairs of environments in each Exp were estimated as the Pearson’s product–moment correlation.

### Genotypic data and analyses

In all Exp, the parental lines were genotyped using whole-genome resequencing. The DNA extraction, library preparation, sequencing and the variant calling pipeline were the same as in Schulthess et al., (2022). The reference genome v2.1 of Chinese Spring (Zhu et al. 2021) was used to map sequencing data. More than 7.5 million SNPs were anchored separately in each Exp. After obtaining preliminary genotype data, further filtering was conducted using vcftools (v0.1.16) (Danecek et al. 2011) to keep high-quality data. Briefly, data quality control for the final dataset used in subsequent analysis included filtering for QUAL ≥ 40 (meaning that the confidence of correct variant call is above 99.99%), read depth for homozygous calls ≥ 10, heterozygosity < 0.05 and minor allele frequency (MAF) ≥ 0.05. Only bi-allelic markers were kept. Markers with missing rate above 30% were removed, and the remaining ones were imputed with beagle (version 5.2) (Browning and Browning 2009). Finally, linkage disequilibrium (LD) pruning was performed with PLINK (v1.9) (Chang et al. 2015) as the squared correlation coefficient (*r*^2^) of 0.9 (Hill and Robertson 1968). In the end, around 640,000 high-quality SNPs remained for each Exp (Supplemental Table S1). The SNP profiles of the hybrids were derived based on the SNP profiles of their homozygous parental inbred lines. The entire analysis was completed in the R environment (v4.1.1) (R Core Team 2021).

### Genome-wide association mapping

In this study, three different models for GWAS were implemented with distinct purposes. Throughout this subsection, the number of genotypes, markers and environments are denoted by n, k and p, respectively. Let ${M}_{a}$ and ${M}_{d}$ be the n×p additive and dominance marker matrices. The F_∞_-metric (Zeng et al. 2005) was used for coding the markers. That is, if the *j*th marker for the *i*th genotype is homozygous for the reference allele (resp. heterozygous, homozygous for the alternative allele), the (i, j) entry in ${M}_{a}$ is coded as 1 (resp. 0, -1) and the (i, j) entry in ${M}_{d}$ is coded as 1 (resp. 0, 1).

In the first model, a standard GWAS was performed to test the main additive and dominance effects of markers across environments within each Exp. Markers with significant main effects across environments are likely indicators for stable QTL. The model is described as follows:

3 $$\begin{array}{*{20}c} {y = 1_{n} \mu + m_{1} a + m_{2} d + g_{a} + g_{d} + \varepsilon ,} \\ \end{array}$$

where y is the n-dimensional vector of BLUEs of all genotypes, including the parental lines and hybrids; ${1}_{n}$ is the n-dimensional vector of ones, μ is the common intercept; a and d are the additive and dominance effects of the marker being tested (modeled as fixed effects); ${m}_{1}$ and ${m}_{2}$ are the n-dimensional additive and dominance coding vectors of the marker; ${g}_{a}$ and ${g}_{d}$ are the n-dimensional vectors of total additive and dominance genetic effects; ${g}_{a}\sim N\left(0,{G}_{a}{\sigma}_{a}^{2}\right)$ and ${g}_{d}\sim N\left(0,{G}_{d}{\sigma}_{d}^{2}\right)$; ${G}_{a}$ and ${G}_{d}$ are the additive and dominance genomic kinship matrices calculated as ${G}_{a}={M}_{a}{M}_{a}{\prime}/{c}_{a}$ and ${G}_{d}={M}_{d}{M}_{d}{\prime}/{c}_{d}$, where ${c}_{a}=\mathrm{m}\mathrm{e}\mathrm{a}\mathrm{n}(\mathrm{d}\mathrm{i}\mathrm{a}\mathrm{g}({M}_{a}{M}_{a}{\prime}))$ (Vitezica et al. 2017); $\varepsilon \sim N\left(0,{{I}_{n}\sigma }_{\varepsilon }^{2}\right)$ is the n-dimensional vector of residuals; and ${I}_{n}$ is the n×n identity matrix. The Wald test was applied to test the significance of $\widehat{a}$ and $\widehat{d}$. Namely, the test statistics were ${T}_{a}={\widehat{a}}^{2}/\mathrm{v}\mathrm{a}\mathrm{r}(\widehat{a})$ and ${T}_{d}={\widehat{d}}^{2}/\mathrm{v}\mathrm{a}\mathrm{r}(\widehat{d})$, both following a Chi-square distribution of one degree of freedom. We also performed a joint test for $\widehat{a}$ and $\widehat{d}$ together. That is, let $\widehat{\alpha }=(\widehat{a},\widehat{d}){\prime}$ and the test statistic was ${T}_{\alpha }=\widehat{\alpha }^{\prime}{\mathrm{c}\mathrm{o}\mathrm{v}(\widehat{\alpha })}^{-1}\widehat{\alpha }$, which follows a Chi-square distribution of two degrees of freedom.

To investigate markers which effects vary significantly across different environments, we tested the additive-by-environment and dominance-by-environment interaction effect for each marker in the second model, taking the genotype-by-environment (G × E) effects into account. The model has the following form:

4 $$\begin{aligned} = & 1_{nk} \mu + 1_{k} \otimes m_{1} a + 1_{k} \otimes m_{2} d + \left[ {\begin{array}{*{20}c} {I_{k - 1} \otimes m_{1} } \\ {0_{{n \times \left( {k - 1} \right)}} } \\ \end{array} } \right]a_{e} + \left[ {\begin{array}{*{20}c} {I_{k - 1} \otimes m_{2} } \\ {0_{{n \times \left( {k - 1} \right)}} } \\ \end{array} } \right]d_{e} \\ & + \;\left( {I_{k} \otimes 1_{n} } \right)e + \left( {1_{k} \otimes I_{n} } \right)g_{a} + \left( {1_{k} \otimes I_{n} } \right)g_{d} + i_{a} + i_{d} + \varepsilon , \\ \end{aligned}$$

where $y=({y}_{11}, {y}_{21}, \dots {y}_{n1}, {y}_{12}, {y}_{22}, \dots {y}_{n2}, \dots , {y}_{1k}, {y}_{2k}, \dots {y}_{nk})$ is an nk-dimensional vector, ${y}_{ij}$ denotes the observed phenotypic record of the *i*th genotype in the *j*th environment, ${1}_{nk}$ is the nk-dimensional vector of ones, μ is the common intercept, a, d, ${m}_{1}$ and ${m}_{2}$ are the same as in Eq. 5, ⊗ denotes the tensor product of two matrices, e is the k-dimensional vector of environmental effects, $e\sim N\left(0,{I}_{k}{\sigma}_{e}^{2}\right)$, and ${a}_{e}$ (resp.${a}_{d}$) is the (k-1)-dimensional vector whose entries are the interaction effect between the additive (resp. dominance) marker effect and the first k-1 environments. The interaction effect for the last environment was not modeled due to collinearity, and ${0}_{n\times (k-1)}$ denotes the n×(k-1) matrix whose entries are all zeros; ${g}_{a}$ and ${g}_{d}$ were the same as in Eq. 3; ${i}_{a}\sim N\left(0,{I}_{k}\otimes {G}_{a}{\sigma}_{a\times e}^{2}\right)$ (resp.${i}_{d}\sim N\left(0,{I}_{k}\otimes {G}_{d}{\sigma}_{d\times e}^{2}\right)$) is the *nk*-dimensional vector of interaction effects between the total additive (resp. dominance) genetic effects and the environments; and *ε*$\sim N\left(0,{{I}_{nk}\sigma }_{\varepsilon }^{2}\right)$ is the nk-dimensional vector of residual effects. The Wald test was applied to test the significance of ${\widehat{a}}_{e}$ and${\widehat{d}}_{e}$. Namely, the test statistics were${T}_{{a}_{e}}={\widehat{a}}_{e}^{\prime}{\mathrm{v}\mathrm{a}\mathrm{r}({\widehat{a}}_{e})}^{-1}{\widehat{a}}_{e}$,${T}_{{d}_{e}}={\widehat{d}}_{e}^{\prime}{\mathrm{v}\mathrm{a}\mathrm{r}({\widehat{d}}_{e})}^{-1}{\widehat{d}}_{e}$, both following a Chi-square distribution of k-1 degrees of freedom. Similar to the first model, a joint test for ${\widehat{a}}_{e}$ and ${\widehat{d}}_{e}$ together was also performed. The test statistic was ${T}_{{\alpha}_{e}}={\widehat{\alpha }}_{e}^{\prime}{\mathrm{v}\mathrm{a}\mathrm{r}({\widehat{\alpha }}_{e})}^{-1}{\widehat{\alpha }}_{e}$, where ${\widehat{\alpha }}_{e}=({\widehat{a}}_{e},{\widehat{d}}_{e}){\prime}$, a $\left(2k-2\right)$-dimensional vector, and it follows a Chi-square distribution of 2k-2 degrees of freedom.

The previous model provides a global test for the interaction effect between the environment and each marker. But it does not tell us the pattern of interaction. In fact, we are interested in the case in which a marker has significant effects in one or several environments, but not in the others. Thus, we applied the third model, in which we tested the additive and dominance effects of all markers within each environment. The model is only slightly different from the second one:

5 $$y = 1_{nk} \mu + (I_{k} \otimes m_{1} )a_{s} + (I_{k} \otimes m_{2} )d_{s} + \left( {I_{k} \otimes 1_{n} } \right)e + \left( {1_{k} \otimes I_{n} } \right)g_{a} + \left( {1_{k} \otimes I_{n} } \right)g_{d} + i_{a} + i_{d} + \varepsilon ,{ }$$

where all notations are the same as in Eq. 4 except that ${a}_{s}$ (resp. ${d}_{s}$) is the k-dimensional vector of within-environment additive (resp. dominance) effects of the marker being tested. That is, ${a}_{s}=\left({a}_{s1},{a}_{s2},\dots ,{a}_{sk}\right){\prime}, {d}_{s}=\left({d}_{s1},{d}_{s2},\dots ,{d}_{sk}\right){\prime}$, and for each $i\in \{\mathrm{1,2},\dots ,k\}$, ${a}_{si}$ (resp. ${d}_{si}$) is the additive (resp. dominance) effect of the marker in the *i*th environment. We again applied the Wald test to test the significance of each ${a}_{si}$ and ${d}_{si}$. The test statistics were ${T}_{{a}_{si}}={\widehat{a}}_{si}^{2}/\mathrm{v}\mathrm{a}\mathrm{r}({\widehat{a}}_{si})$, ${T}_{{d}_{si}}={\widehat{d}}_{si}^{2}/\mathrm{v}\mathrm{a}\mathrm{r}({\widehat{d}}_{si})$, both following a Chi-square distribution of one degree of freedom. As in the previous two models, we performed a joint test for ${a}_{si}$ and ${d}_{si}$ together with the test statistic ${T}_{{\alpha}_{si}}={\widehat{\alpha }}_{si}^{\prime}{\mathrm{v}\mathrm{a}\mathrm{r}({\widehat{\alpha }}_{si})}^{-1}{\widehat{\alpha }}_{si}$, where ${\widehat{\alpha }}_{si}=({\widehat{a}}_{si},{\widehat{d}}_{si}){\prime}$, following a Chi-square distribution of two degrees of freedom.

The three GWAS models were implemented by in-house-developed R scripts. For each model, we followed the P3D approach (Zhang et al. 2010). Variance components were estimated only once in the null model (i.e., the model without including any marker to be tested) and fixed throughout the test for all markers. All LMMs were solved by using the R package ASReml-R (Gilmour et al. 1995). The genome-wide threshold for the *p* values was determined as *p* < 0.05 after Bonferroni correction for multiple testing (Dunn 1961).

### The delineation of quantitative trait loci

Main effects as well as effects specific for certain environments, regardless whether additive, dominance or joint effects that had been assessed in the GWAS model were considered for QTL delineation. MTAs were grouped into QTL based on LD between markers using the clump function in PLINK (v1.9) (Chang et al. 2015) for a particular Exp. Significant markers located on the same chromosome and within 50 Mbp were considered as representatives of the same QTL when their LD measured as ${r}^{2}$ was greater than 0.3.

LD between all significant SNPs which were identified for a particular Exp was calculated. Values larger than 0.7 for SNPs located on different chromosomes indicated few significant SNPs which had been assigned to the wrong chromosome region. These were omitted from further analyses. Significant SNPs assigned to the unknown chromosome but in high LD (*r*^*2*^ > 0.7) to QTL regions on the 21 wheat chromosomes were deemed redundant and removed.

Independent QTL from different Exp sharing at least one MTA were merged. If two independent QTL were located on the same chromosome within a 50 Mbp window but did not share any MTAs, further evaluation was required: If there is one MTA from a QTL in the first Exp that was present in the second Exp but not significant, the average *r*^2^ was calculated between this (nonsignificant) MTA and all MTAs in the second QTL, using the data from the second Exp. Such procedure was repeated for all such MTA. If the final averaged *r*^*2*^ > 0.7, the two QTL were merged.

QTL which were identified in a single environment and/or by main effects were termed single-environment QTL (SEQ), those that were found in at least two environments multi-environment QTL (MEQ).

The degree of dominance (*d/a*) for each QTL, represented by the lead SNP, was calculated according to a previous study (Cui et al. 2023). To better account for the characteristics of disease resistance traits, the absolute values of additive effects were used in the ratio (*d/|a|*). The thresholds were defined as follows: *d/|a|*> 0.2, indicating positive dominance effects; − 0.2 ≤ *d/|a|*≤ 0.2, classified as additive; − 0.8 ≤ *d/|a|*< − 0.2, indicating partial dominance; − 1.2 ≤ *d/|a|*< − 0.8, classified as complete dominance; and *d/|a|*< − 1.2, indicating overdominance. Negative degrees of dominance indicate resistance.

The phenotypic variance explained (PVE) for each QTL, represented by the lead SNP, was estimated using linear models in R (v4.1.1) (R Core Team 2021). The PVE for the additive effect was calculated by fitting a model including only the additive genotype coding vector. The total PVE was estimated by fitting a model including both the additive and dominance genotype coding vectors. Subtracting the additive PVE from the total PVE resulted in the PVE for the dominance effect.

### Identification of candidate genes

All annotated high-confidence genes mapping within the intervals specified by the different MEQ were extracted from the Chinese Spring reference genome (v2.1; Zhu et al. 2021). Gene function annotation was downloaded from Triticeae-Gene Tribe (http://wheat.cau.edu.cn/TGT/, (Chen et al. 2020). The list of confirmed *NLR* genes included 1,827 gene models encompassing a P-loop, a minimum of three consecutive motifs of the NB-ARC domain and one or more motifs associated with LRRs (Steuernagel et al. 2020). ANNOVAR (Wang et al. 2010) was used to annotate sequence variants in gene regions including untranslated regions, exons and introns. Additionally, it was differentiated between SNPs present in 2 kbp intervals immediately upstream of the transcription start site and downstream of the transcript terminus of a gene and the remainder of the intergenic regions. Genes showing nonsynonymous SNPs in exons were prioritized as candidate genes.

MEQ regions that had been detected across Exp were extended 0.5 Mbp upstream and downstream and all SNPs (without LD pruning) within these regions rescanned using the joint test to establish their *p* values. Significant SNPs were subjected to SNP annotation analysis as detailed above.

## Results

### Strong genotype-by-environment interactions

**Fig. 1:**
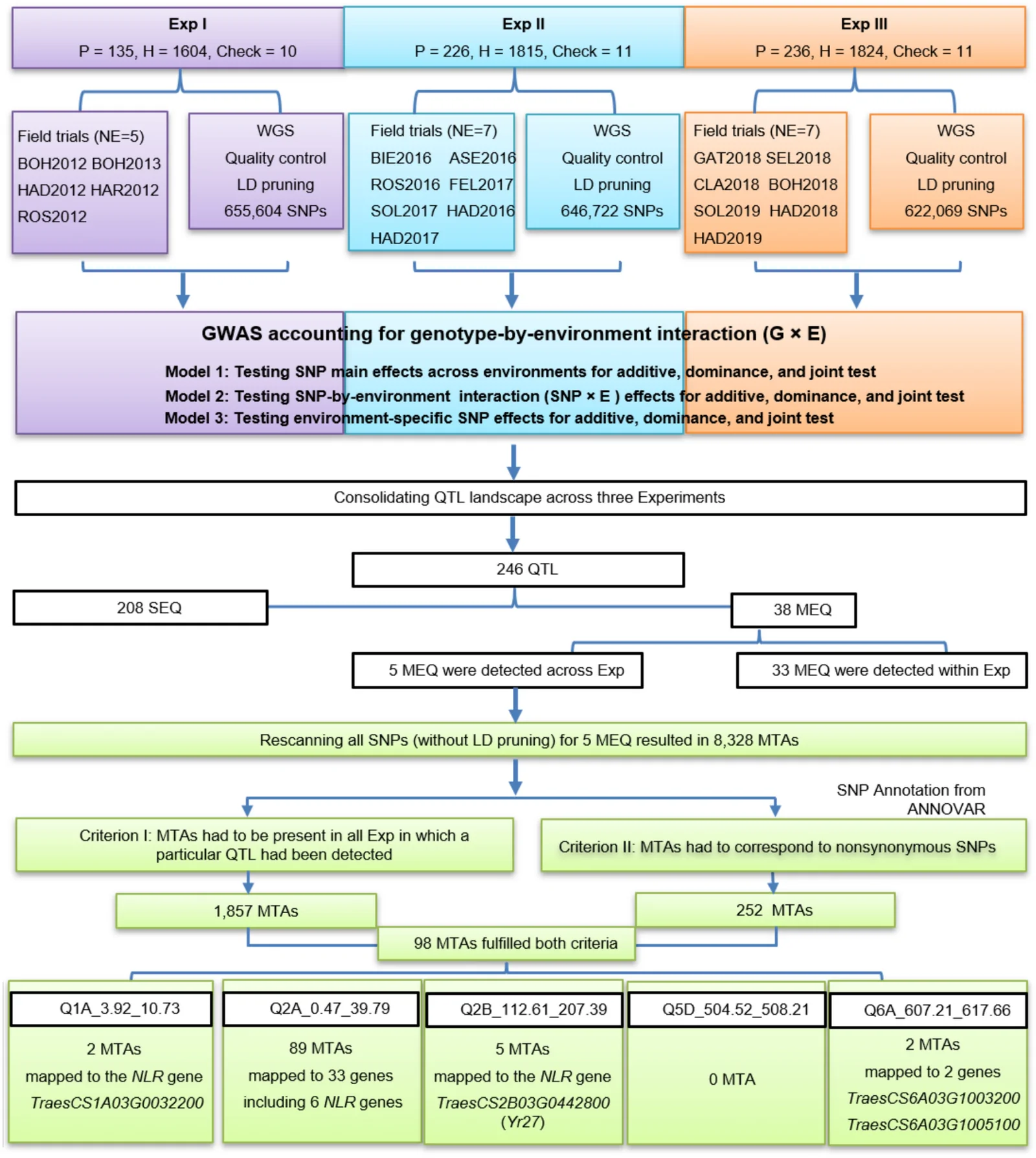
A brief summary of the experimental design, strategy of data analysis and key findings of this study shown as a workflow chart. Exp: experimental series; P: the number of parental lines; H: the number of hybrids; Check: the number of common checks evaluated in each environment; E: the number of environments; WGS: whole-genome sequencing; LD: linkage disequilibrium; SNP: single-nucleotide polymorphism; GWAS: genome-wide association study; MTA: marker–trait association; QTL: quantitative trait loci; MEQ: multi-environment QTL; SEQ: single-environment QTL

**Fig. 2:**
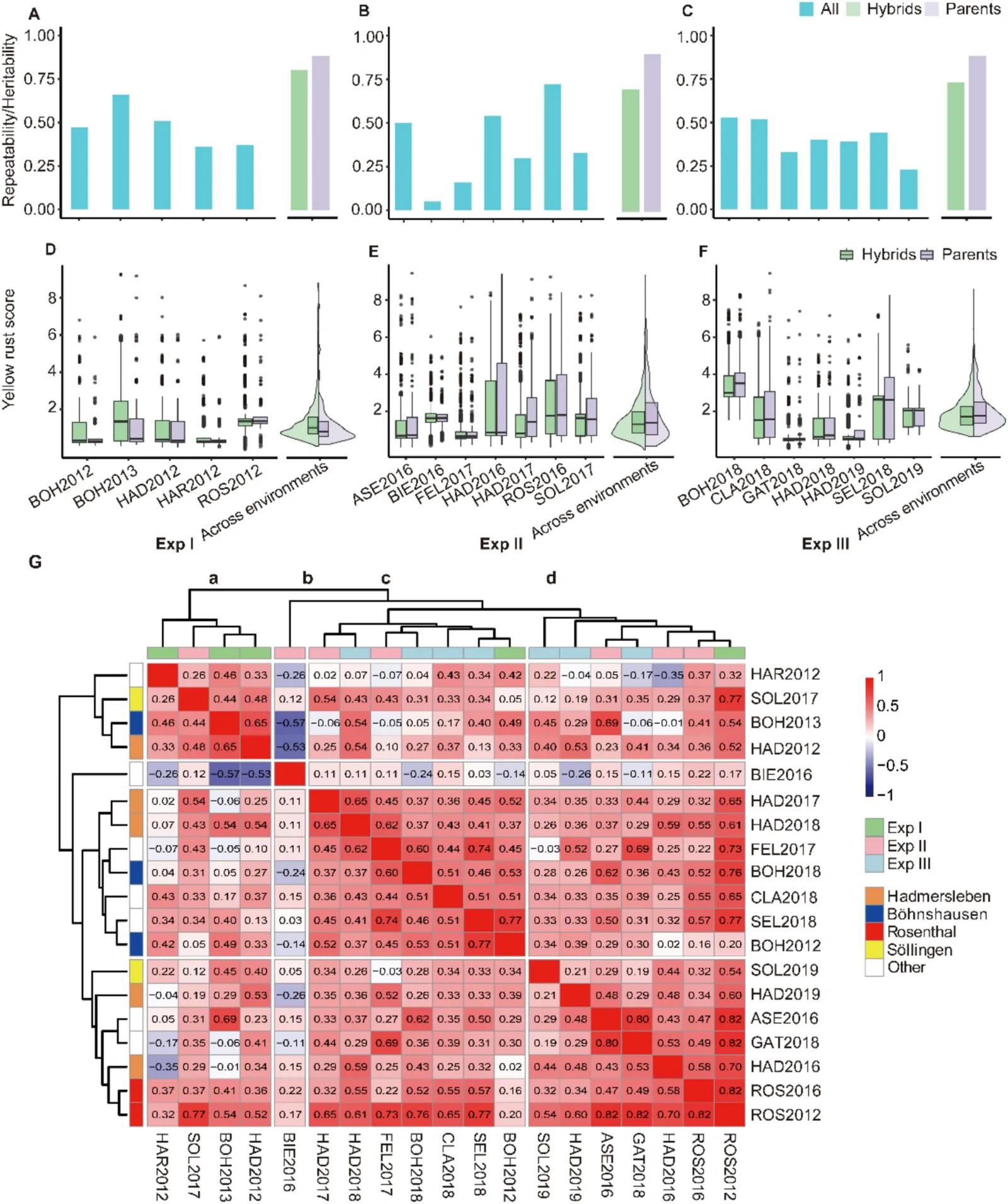
Characteristics and inter-environment relationships of phenotypic data for YR severity across environments. **A**–**C** The genomic repeatability within each environment and the broad-sense heritability across environments. (**D**–**F**) The distribution of YR severity scores (1, fully resistant; 9, fully susceptible) within each environment and the best linear unbiased estimations (BLUEs) across environments. Boxes enclose 50% of the central data, including the median (horizontal black bold line), while whiskers extend to ± 1.5 times the interquartile range. Details of the environments are given in Supplemental Table S2. **G** A hierarchical clustering of 19 environments in the three Exp based on the pairwise Pearson correlation coefficients for YR severity. Positive and negative correlations were indicated according to the red and blue color gradient, respectively. For each environment, colored boxes indicate the corresponding Exp and location above and to the left of the correlation table, respectively. All color keys are shown at the right. Environments are described in Supplemental Table S2 and were classified according to the hierarchical clustering into four groups (a-d)

In multi-environment trials nested within three experimental series, we phenotyped 5,243 hybrids and their 597 parents for YR severity at the adult plant stage (Fig. 1 and Supplemental Table S1). In total, 19 environments were tested (Supplemental Table S2). Different genotype groups were assessed in each Exp, with a minimum of nine common genotypes serving as bridge (Supplemental Table S3). The estimates of genomic repeatability (Fig. 2A–C) were predominantly higher than 0.3 for most environments, with three exceptions: BIE2016 (0.05), FEL2017 (0.17) in Exp II and SOL2019 (0.23) in Exp III. Nevertheless, the broad-sense heritability exceeded 0.7 in all three Exp. YR severity scores were widely distributed across environments in each of the three Exp. Notably, in Exp I, genotypes showed overall greater resistance to YR compared to those in the other Exp (Fig. 2D–F).

To investigate *G* × *E* interactions, we estimated pairwise correlations between YR severity of different environments and conducted hierarchical cluster analysis. This revealed four groups (Fig. 2G). Group (b) comprised only environment BIE2016, which exhibited the lowest genomic heritability. The other three groups included environments from two or all three Exp. The correlation between environments across different groups ranged from −0.57 to 0.77. The correlations within each group also varied widely: Group (a) showed a range from 0.26 to 0.65, Group (c) from 0.36 to 0.77 and Group (d) from 0.19 to 0.82. These results underscore the large impact of *G* × *E* interactions on YR severity.

Next, the similarity between environments defined by the same location but in different years was assessed (Fig. 2G). Data for the location Hadmersleben (HAD) were available for five years. Notably, these five environments were classified into three distinct groups: one in Group (a) and two each in Groups (c) and (d). The pairwise correlations among the five environments varied between 0.25 and 0.65. Similarly, the three environments analyzed for the location Böhnshausen (BOH) were assigned to two groups (correlations 0.05–0.53) and the two poorly correlated (0.12) environments in Söllingen (SOL) did not belong to the same group either. These results suggest that the interaction effects involving the year effect, i.e., genotype-by-year (G × Y) and/or genotype-by-location-by-year interactions, are an important component in the overall G × E interaction.

### QTL consistency was low across experimental series

**Fig. 3:**
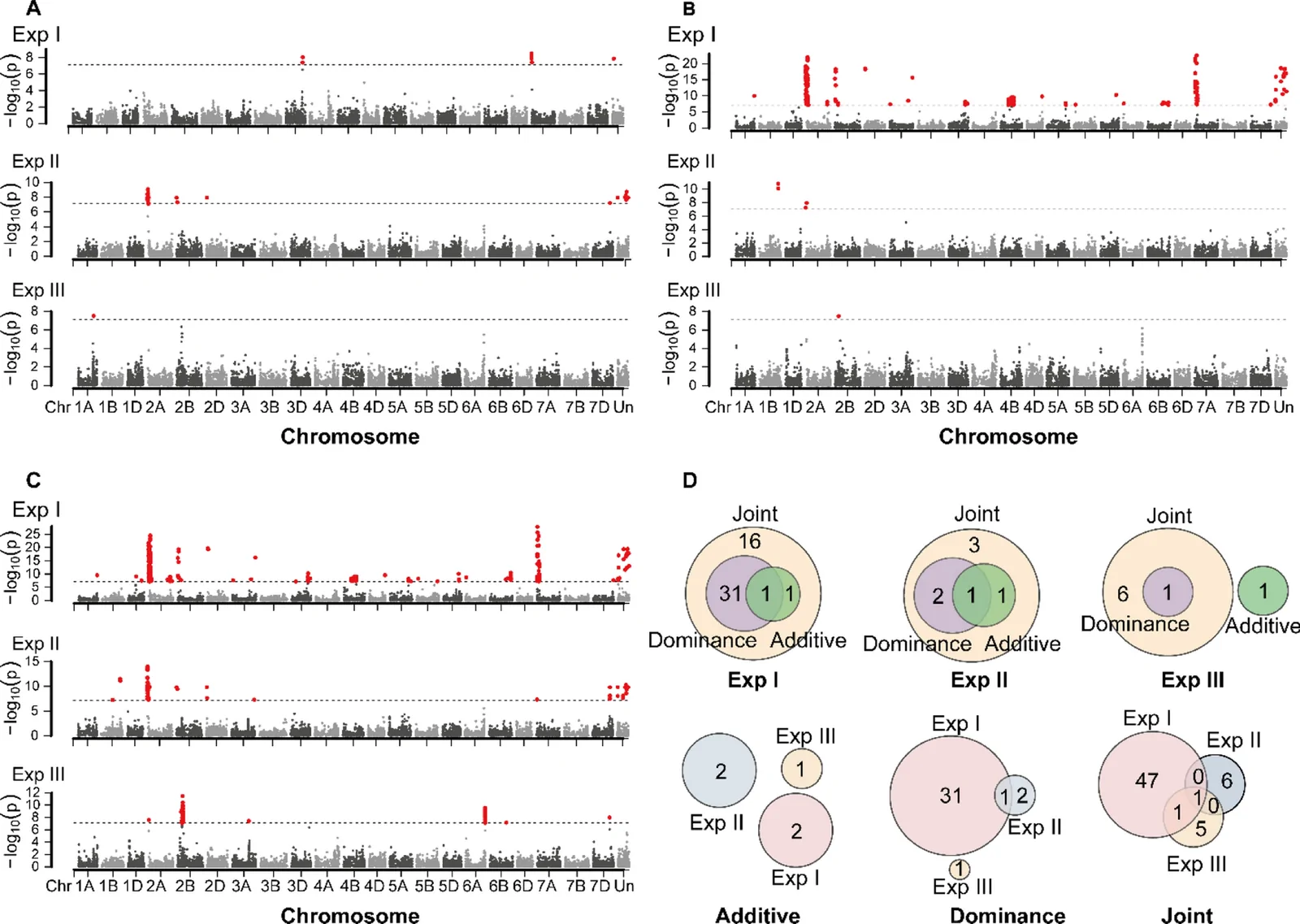
Results of GWAS across environments within each Exp for additive, dominance and a joint test of both effects. Manhattan plots showing the results of a GWAS study for additive main effects (**A**), dominance main effects (**B**) and a joint test of additive and dominance main effects (**C**) across environments within each Exp. In each panel, the genome-wide threshold of *p* < 0.05 after Bonferroni correction was indicated by the stippled horizontal line. **D** The number of main-effect QTL identified for additive, dominance and a joint test of additive and dominance effects in each Exp

We initially focused on YR severity estimated across environments to detect QTL main effects, considering additive and dominance effects separately. More significant MTAs were detected in Exp I compared to the other Exp (Fig. 3A and B). A joint test of additive and dominance effects increased the number of MTAs in all Exp (Fig. 3C).

Subsequently, we grouped all MTAs into QTL confirming the previous finding at the level of QTL (Fig. 3D, Supplemental Table S4). The overlap between QTL exhibiting significant additive and dominance effects was minimal, with no overlap at all for Exp III. In Exp I, we identified many more QTL with significant dominance effects (32) compared to additive effects (2), a pattern not observed in Exp II and Exp III. Even when using the more powerful joint test only one QTL in common between all three Exp (Q2A_0.47_39.79) was identified. Note that although all parental lines of the three experimental series belong to the Central European elite breeding pool, only a limited number of common genotypes were evaluated across experimental series. Thus, in addition to the pronounced G × E interactions, differences in the genetic makeup may also explain the low number of common QTL across experimental series.

### Substantial QTL-by-environment interactions

**Fig. 4:**
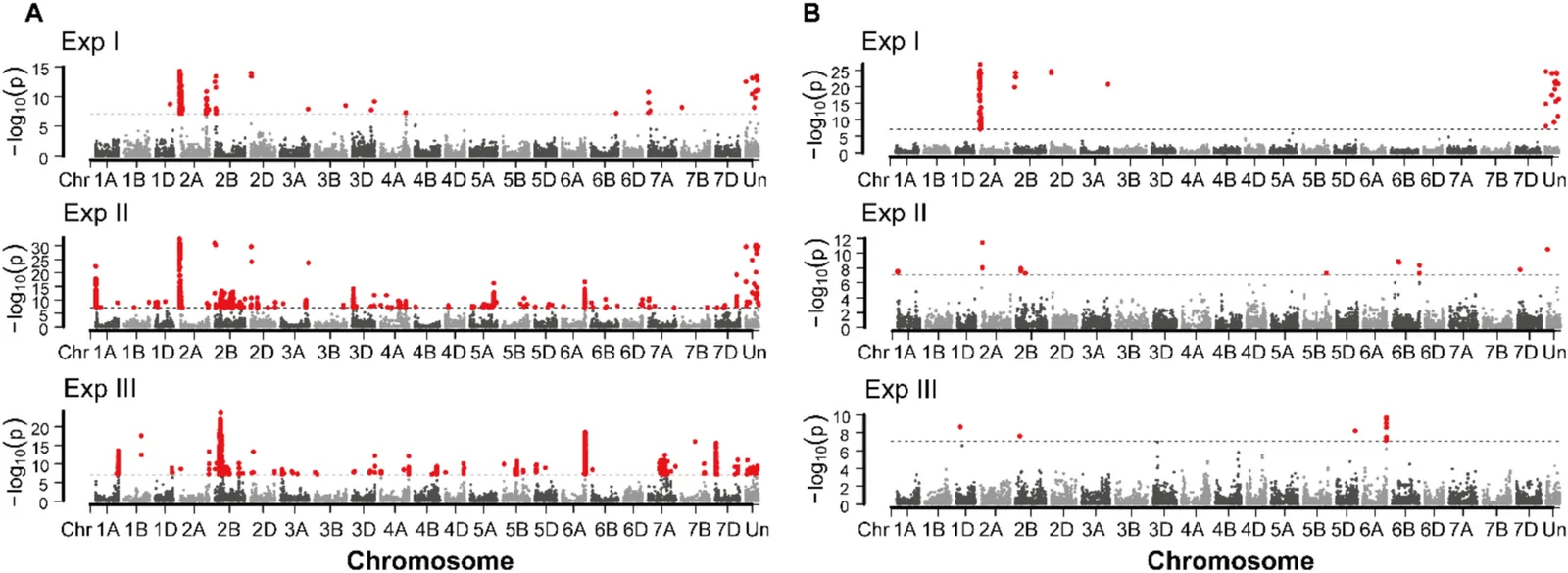
Results of GWAS for marker-by-environment interaction effects within each Exp. Manhattan plots showing the results of GWAS study for additive-by-environment interaction effects (**A**) and dominance-by-environment interaction effects (**B**) within each Exp. In each plot, the genome-wide threshold of *p* < 0.05 after Bonferroni correction was indicated by the stippled horizontal line

Based on the YR scores within environments, we tested interaction effects between markers and environments to identify environment-specific MTAs. The analyses revealed a greater number of markers with significant *p* values under the joint test for interaction effects between markers and environments in Exp II (1,797, −log_10_(*p*) > 7.12) and Exp III (1,123, −log_10_(*p*) > 7.09) compared to Exp I (234, −log_10_(*p*) > 7.12) (Supplemental Table S5). Interaction effects between markers and environments were observed for all wheat chromosomes, with prominent peaks on chromosomes 1A, 2A, 2B and 6A (Supplemental Fig. S1). A comparison of the interaction effects between markers and environments and the MTAs showing main effects revealed a large proportion of shared associations in Exp I and Exp III, whereas in Exp II the interaction effects clearly outnumbered the MTAs (Supplemental Fig. S2A). Importantly, we detected more additive-by-environment compared to dominance-by-environment effects (Fig. 4). Additive interaction effects between markers and environments outnumbered the main additive effect MTAs, whereas the opposite was observed for dominance effects (Supplemental Fig. S2B), suggesting that additive effects are particularly sensitive to environmental variation.

**Fig. 5:**
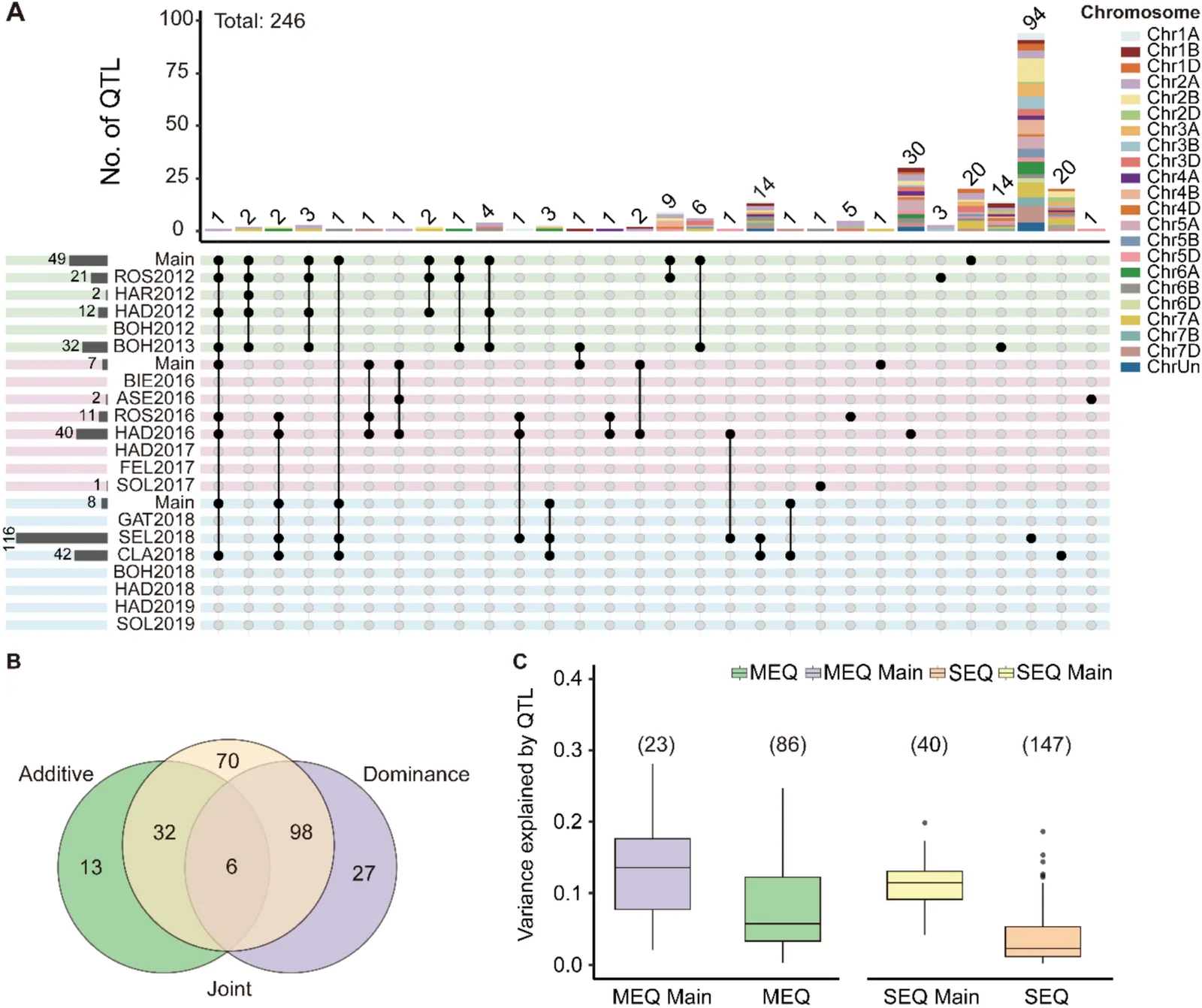
Summary and classification of QTL with significant main and/or environment-specific effects across three Exp. **A** A summary of QTL with significant environment-specific effects under the additive, dominance and joint test. Environments in different Exp were indicated in distinct colors (green, Exp I; pink, Exp II; blue, Exp III). A black dot indicated that a QTL was identified in a specific environment or that it had a significant main effect across environments. The stacked bar graph above shows the number of QTL exhibiting the same pattern. QTL on different chromosomes were indicated according to the color key shown to the right. **B** Most of the 246 QTL were detected under the joint test. QTL with additive and/or dominance effects were not as frequent. **C** Distribution of PVE by MEQ and SEQ depicted for the joint test considering additive and dominance effects. MEQ: multi-environment QTL. SEQ: single-environment QTL. MEQ/SEQ Main: the subset of MEQ/SEQ which was identified by the main effect across environments. The number of statistical values for each box plot is shown in parentheses above the corresponding plot

Genomic regions associated with YR resistance across multiple environments are important targets for breeding efforts. Thus, we evaluated the MTAs within each environment and examined their stability (Supplemental Figs S3─S5). For QTL delineation, all main and environment-specific effects were considered which had been identified in the three Exp, including additive, dominance and joint effects (Supplemental Table S4). Out of the total of 246 QTL, 208 and 38 represented SEQ and MEQ, respectively (Figs. 1, 5A and B). Two recent studies reported 331 and 431 YR QTL (Cheng et al. 2024; Wu et al. 2025). Comparing these with the QTL identified in this study revealed colocalization for 107 out of 208 SEQ (51.44%) and 26 out of 38 MEQ (68.42%) (Supplemental Table S6).

Joint effects detected most QTL (83.7%) (Fig. 5B), with MEQ (97.4%) being identified at a higher frequency than SEQ (81.3%). Dominance effects detected more QTL (53.3%) than additive effects (20.7%) (Fig. 5B). Only five QTL were found for two or three Exp, four (Q1A_3.92_10.73, Q2B_112.61_207.39, Q5D_504.52_508.21, Q6A_607.21_617.66) were detected in Exp II and Exp III, and MEQ Q2A_0.47_39.79 was identified in all three Exp. Assessment of the PVE by the QTL revealed significantly (*p* < 0.001, *t*-test) higher values for MEQ than SEQ. Those QTL for which main effects have been detected revealed significantly (*p* < 0.001, *t*-test) larger PVE than those which had solely been detected in specific environments (Fig. 5C and Supplemental Fig. S6A). As many major resistance genes show complete dominance when exposed to specific isolates, the degree of dominance was estimated for our field trials, which are likely to be characterized by a mixture of different *Pst* races. As exemplified for the data obtained with the joint test, most QTL within but also across environments showed negative degrees of dominance indicating resistance in our study, with a large proportion revealing complete dominance and overdominance (Supplemental Fig. S6B and Table S4).

### QTL-by-year interactions indicate potential breakdown of resistance genes

**Fig. 6:**
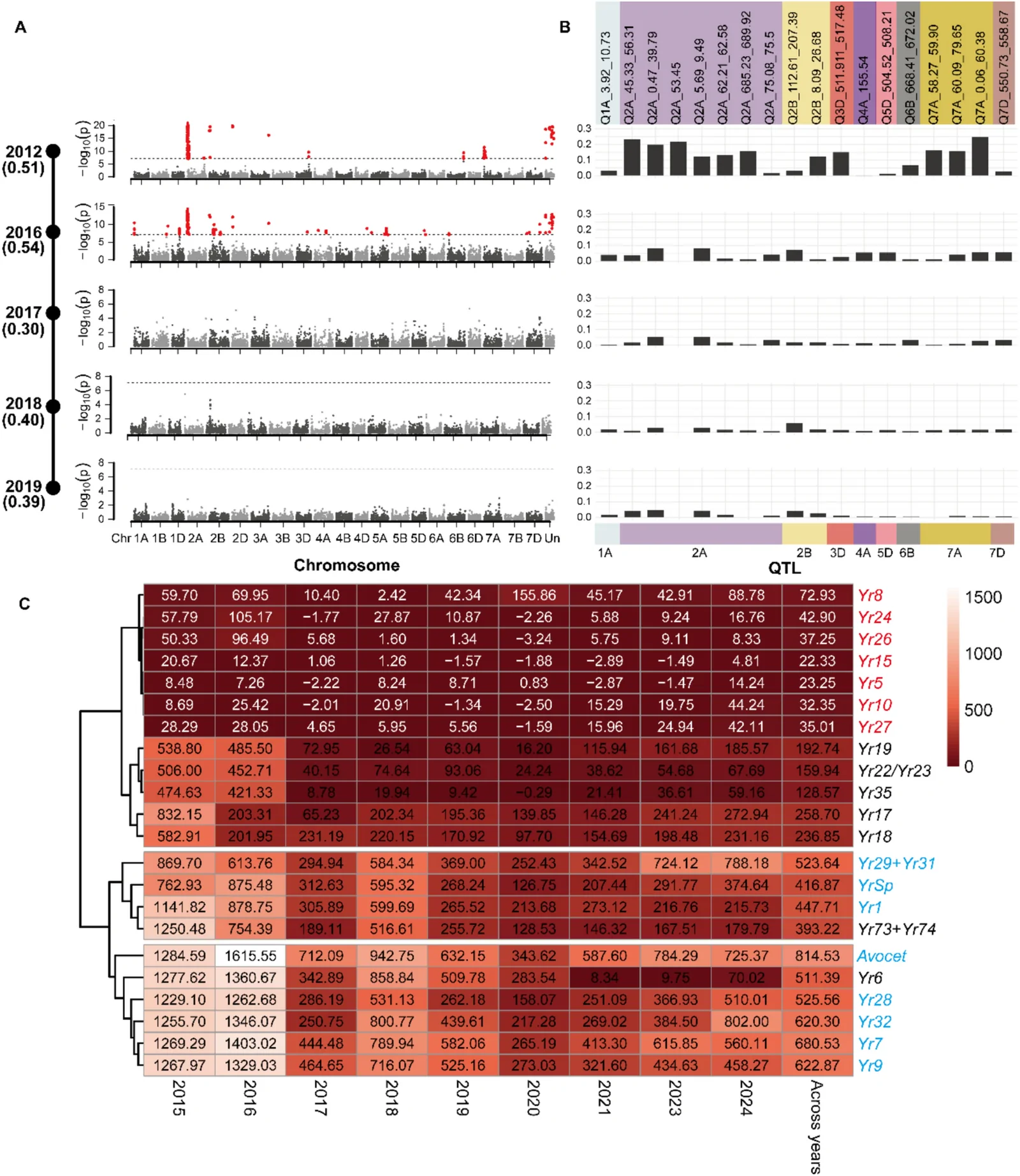
QTL × year interactions identified by GWAS and validated using near-isogenic lines carrying different *Yr* genes across nine years. Manhattan plots showing the results of a GWAS study (**A**) and the proportions of PVE by 18 MEQ (**B**) for YR resistance adopting the joint test of additive and dominance effects for five years and the location Hadmersleben. The genome-wide threshold of *p* < 0.05 after Bonferroni correction was indicated by the dashed horizontal line in each of the plots. The genomic heritability for each individual environment is shown in parentheses beneath the year. **C** Levels of yellow rust (YR) infection of the susceptible wheat cultivar ‘Avocet’ and 21 genotypes carrying different YR resistance genes (*Yr*) across nine years. The severity of YR infection was measured as the area under the disease progress curve (AUDPC, Moll et al. 1996). Three genotypes each carried two *Yr* genes (*Yr22* + *Yr23*, *Yr29* + *Yr31*, *Yr73* + *Yr74*) and 18 genotypes each carried one of the following genes: *Yr1*, *Yr5*, *Yr6*, *Yr7*, *Yr8*, *Yr9*, *Yr10*, *Yr15*, *Yr17*, *Yr18*, *Yr19*, *Yr24*, *Yr26*, *Yr27*, *Yr28*, *Yr32* (*YrCV*), *Yr35* and *YrSP*. Levels of infection were evaluated in four replicated field trials over nine years (2015–2024). The AUDPC for each trial was calculated based on estimated percentages of infected leaf area at multiple time points. Genotypes showing consistently high resistance were highlighted in red, while those displaying susceptibility across all years were marked in blue

Next, we analyzed which QTL repeatedly contributed to YR resistance. The results from the Hadmersleben location were examined in detail, since data were available for five years (Fig. 6A and B). SEQ (20) were only observed in 2016, whereas 18 MEQ were identified in 2012 (Exp I) and/or 2016 (Exp II). For these two years, higher genomic heritabilities were noted than in subsequent years in which significant associations were not observed. Differences between years are also reflected in the phenotypic variance explained by the MEQ across the five years. Only MEQ Q2A_0.47_39.79 on chromosome 2A showed significant associations in 2012 and 2016. Significant marker–trait associations mapping to QTL Q2A_0.47_39.79 were detected for all three Exp (Supplemental Table S4), but in Hadmersleben for only two out of the three series, differences between the three experimental populations are therefore not sufficient to explain the observed YR patterns in Hadmersleben. MEQ Q1A_3.92_10.73, Q2B_112.61_207.39 and Q6A_607.21_617.66 were detected in Exp II and Exp III, but in Hadmersleben all three were only observed in year 2016. Whereas the former two QTL were identified based on joint effects (Fig. 6A and B), detection of the latter relied on additive effects (Supplemental Table S4). Significant MTAs mapping to QTL Q1A_3.92_10.73 and Q6A_607.21_617.66 revealed comparable minor allele frequencies in all three experimental series, but QTL were only observed in Exp II and Exp III. In contrast, MEQ Q2B_112.61_207.39 showed a considerably lower allele frequency in Exp I when compared to the other two series in which significant associations were seen for this QTL (Supplemental Table S7).

To further validate the identified QTL-by-year interactions and assess the performance of *Yr* QTL across different years, we evaluated the susceptible cultivar ‘Avocet’ alongside 21 lines carrying different resistance loci in Quedlinburg, Germany, from 2015 to 2024 and estimated the area under the disease progress curve (AUDPC, Moll et al. 1996). The phenotypic data showed a heritability of 0.84. Among the 22 tested genotypes, seven (those carrying *Yr5*, *Yr8*, *Yr10*, *Yr15*, *Yr24*, *Yr26* and *Yr27*) showed consistently high resistance across all years with a maximum AUDPC of 97 in all but two cases, while eight genotypes (those carrying *Yr1*, *Yr7*, *Yr9*, *Yr28*, *Yr29* + *Yr31*, *Yr32*, *YrSp* and ‘Avocet’) displayed susceptibility with minimum AUDPC above 200 except two cases. Lines carrying *Yr17* and *Yr18* were susceptible in 2015 with an AUDPC of 832.15 and 582.91, respectively. In subsequent years values between 65 and 280 were observed (Fig. 6C). This result supports our observation that the *Yr* gene is involved in the pronounced year effect.

### Identification of candidate genes

A first candidate gene search involved all 38 MEQ and the corresponding 1,201 MTAs detected by the joint test (Supplemental Table S7). All QTL regions together spanned 5,100 annotated high confidence genes (Zhu et al. 2021). In the wheat genome, 1,827 genes have been classified as confirmed *NLR* genes (Steuernagel et al. 2020), and 171 of these were present in the MEQ regions. Of all 38 MEQ, 17 showed MTAs in gene regions which included in this study the 2 kbp regions upstream and downstream of the transcribed regions. In total, 179 MTAs mapped to 117 gene regions but only 19 MTAs mapping to seven MEQ corresponded to nonsynonymous SNPs. The 18 genes tagged by nonsynonymous SNPs, including three *NLR* genes, were prioritized as candidate genes. Notably, one *NLR* gene, *TraesCS2B03G0442800*, corresponds to *Yr27* on chromosome 2B (Athiyannan et al. 2022). The other two, *TraesCS2A03G0062000* and *TraesCS2A03G0062300*, were located in MEQ Q2A_0.47_39.79 for which eight candidate genes were identified.

The five MEQ which were detected in more than one Exp were subjected to a detailed search involving all single-nucleotide polymorphisms (SNPs) in the QTL regions (Fig. 1, Supplemental Table S8). Rescanning all SNPs using the joint test resulted in 8,328 MTAs compared to 800 with LD pruning. Likewise, a much larger number of genes showing nonsynonymous exchanges were detected if all SNPs were considered, 73 instead of 12. The prioritization of candidate genes followed a stepwise procedure. As a first step, only the 1,857 significant associations that were present in all Exp in which a particular QTL had been detected were considered (Criterion I, Fig. 1). Shared significant associations were not observed for the two environments in which MEQ Q5D_504.52_508.21 had been observed and all six MTAs assigned to this QTL region mapped to intergenic regions. This MEQ was therefore not analyzed further. As a second step, the MTAs had to correspond to nonsynonymous SNPs, 252 in total (Criterion II, Fig. 1). Only 98 out of 8,328 fulfilled both criteria. For MEQ Q1A_3.92_10.73, two significant associations mapping to the *NLR* gene *TraesCS1A03G0032200* fulfilled both criteria for candidate gene nomination. In case of Q2B_112.61_207.39, five nonsynonymous SNPs also pinpointed a single gene (*Yr27*, *TraesCS2B03G0442800*). *TraesCS6A03G1003200* and *TraesCS6A03G1005100* encoding proteins with LRR domains were tagged by one nonsynonymous SNP each mapping to MEQ Q6A_607.21_617.66. For QTL Q2A_0.47_39.79, the 89 selected MTAs mapped to 33 candidates, including six *NLR* genes (*TraesCS2A03G0058600*, *TraesCS2A03G0058700*, *TraesCS2A03G0059300*, *TraesCS2A03G0059900*, *TraesCS2A03G0062000* and *TraesCS2A03G0062300*).

## Discussion

### Need for characterization of *Pst* populations in field trials to dissect QTL × E

As basis of our data mining strategy, we implemented a systematic GWAS framework based on linear mixed models (LMMs) to test additive, dominance and joint effects for all markers, both within and across environments, as well as their interaction effects with the environments. As shown here for YR in wheat, this refined method offers a powerful tool for dissecting the temporal and spatial dynamics of the genetic architecture of complex traits. While few previous studies have also explored the QTL × *E* interactions using LMMs, there are important differences in model structure between their approaches and ours. For example, some models explicitly incorporated environmental covariates (Sul et al. 2016; Li et al. 2021; Zhong et al. 2023), whereas no such covariates were available in our study. Other studies modeled QTL × *E* interactions similarly to our approach but did not account for interactions between genetic background and environment (Moore et al. 2019; Sakai et al. 2023). Related approaches include modeling environment effects as fixed covariates rather than as a random vector (Branchereau et al. 2023), or capturing genetic background × environments interactions within the residuals rather than as a separate random term (Malosetti et al. 2013). With the exception of the latter study, most previous studies considered only additive QTL effects and their additive-by-environment interactions. Our models are closely aligned with the base models implemented in the software package 3VmrLMM (Li et al. 2022), which can assess QTL, QTL × *E* and QTL × QTL effects. However, because modeling QTL × QTL interactions is computationally demanding, a two-step procedure was established to reduce the computational load. As our study did not include QTL × QTL interactions, test statistics for QTL main effects and QTL × *E* interactions were computed directly from the fitted model.

Applying the developed GWAS framework, QTL × *E* patterns for loci associated with YR severity were evaluated in 19 environments. Lack of QTL stability due to interaction effects with locations was observed in all three Exp, as only a subset of the environments analyzed in a particular Exp revealed QTL. In accordance with other reports (e.g., Rollar et al. 2021), the benefit of analyzing individual environments in addition to main effects was therefore clearly evident in our analysis. The majority of MEQ that were detected in a particular Exp were identified in just seven environments. This readily explains why trait values showed varying correlation levels when different environments of a certain Exp were compared and why only 55% of the MEQ were detected based on main effects. For the locations Böhnshausen and Hadmersleben, QTL were found for only one out of two environments analyzed in different years of a particular Exp, demonstrating interaction effects with years. Evidence for pronounced year effects was also observed for the analysis of lines carrying introgressed *Yr* loci. QTL stability may be influenced by pathogen isolates and/or environmental variables. For example, temperature influenced YR infections in wheat (Milus et al. 2015). However, our study did not allow to discriminate between the effects of specific environmental variables. In future, it will be crucial to integrate environmental factors and YR race tracking (Li et al. 2023) for deciphering the genetic architecture of disease resistance traits.

### Stringent candidate gene selection

The number of genotypes studied here is substantially larger than those evaluated in other GWAS studies focusing on resistance to YR in Central European winter wheat elite lines (Beukert et al. 2020; Miedaner et al. 2020). Compared to earlier studies which relied on SNP arrays (Beukert et al. 2020) or genotyping by sequencing (Miedaner et al. 2020), a more than 25-fold higher SNP density was achieved here by employing whole-genome resequencing, even when considering SNPs after filtering. The larger panel size together with the very high marker number allows for a detailed study of the genetic architecture of the trait of interest. Similar to the results reported here for Central European elite cultivars and their corresponding hybrids, several hundred QTL each were reported in GWAS studies in which the Watkins landraces (Cheng et al. 2024) and a panel of worldwide accessions (Wu et al. 2025) were analyzed. Many QTL were in common between the studies. For example, two-thirds of the MEQ colocalized with QTL described in the other two studies. Nonetheless, the need for multiple large-scale GWAS studies is evident, as many QTL were also specific for each of the three panels.

Our study is based on association mapping in hybrid populations. The number of parental lines constrains both the extent of recombination and the diversity of independent local haplotypes in the hybrid population. However, a key advantage of using hybrid populations is the ability to test haplotypes across multiple genetic backgrounds, effectively increasing the sample size and enhancing the power to detect QTL compared to analyses based solely on parental lines. Consistent with this expectation, we identified five promising QTL regions across multiple experiments. Another advantage of using a hybrid population is the opportunity to estimate the degree of dominance. Most QTL, both within and across environments, exhibited a desired negative degree of dominance, with a significant percentage demonstrating complete dominance and overdominance (Supplemental Fig. S6B and Table S4). Therefore, it is enough to fix the favorable resistance allele in one parental pool. This minimizes breeding efforts and saves resources.

By integrating functional annotation of sequence variants, we were able to home in on candidate genes at an unprecedented level of resolution for QTL that were detected in multiple Exp (Fig. 1). First, causative SNPs should be present in all Exp in which a particular QTL had been detected. This criterion exploited the different LD structures in the three GWAS panels to delineate QTL regions more precisely by selecting common MTAs. Second, MTAs had to correspond to nonsynonymous SNPs. This criterion required whole-genome resequencing data and an annotated reference genome. Together, the strict criteria proved effective in reducing the number of MTAs, but could have increased the rate of false negatives. For four MEQ that shared significant associations in more than one panel, 37 candidate genes were nominated, eight of these corresponded to *NLR* genes. We focused in our study on adult plant stage resistance (APR), not seedling stage resistance. Screening of seedlings is known to miss many APR-associated resistance mechanisms, which are often governed by quantitative traits rather than single R genes. However, resistance conferred by *NLR* genes can also be expressed during the adult plant stage and thus may be captured in APR phenotyping assays. Consequently, the detection of *NLR*-encoding genes in our study is not unexpected and aligns with the known functional overlap between *R* gene-mediated resistance and APR. However, the presence of effective race-specific resistance genes may also mask (partially) APR-associated resistance genes present in the same genotypes. This could reduce the power to detect QTL and compromise our data mining strategy that focused on MTAs consistently detected across experimental series.

Notably, one or two candidate genes per QTL resulted for three of the MEQ, paving the way for further functional validation. The adequacy of the adopted strategy is evident as one of the nominated candidate genes (*TraesCS2B03G0442800*) corresponds to *Yr27*, a characterized YR resistance gene (Athiyannan et al. 2022). In contrast, MEQ Q2A_0.47_39.79 showed on average an approximately threefold higher density of MTAs in annotated gene regions than the other three and many candidate genes (33) were identified, likely reflecting the tight cosegregation of genes mapping to the introgression region in the association panels.

Exclusively focusing on coding region variants is not appropriate for the identification of variants that modulate gene expression levels. To account for this shortcoming, all SNPs in gene regions, but excluding synonymous SNPs, rather than only nonsynonymous substitutions should be considered. Importantly, adopting such a less stringent approach also efficiently reduced the size of the QTL regions and revealed five, 17 and 33 MTAs that corresponded to one, eight and nine candidate genes for three of the MEQ (Supplemental Table S8, MEQ Q1A_3.92_10.73, Q2B_112.61_207.39, Q6A_607.21_617.66), but not for the MEQ which coincided with an introgression region (Q2A_0.47_39.79).

Chinese Spring served as a reference genome for SNP calling and mapping in this study, but if needed, another—potentially more suitable—reference genome could be integrated in the analysis (Kale et al. 2022). Compared to RenSeq of association panels, whole-genome resequencing is more cost-intensive. However, as shown in this study, if implemented for hybrid panels, GWAS based on resequencing can be conducted even in wheat for several thousand individuals.

### *Yr* QTL in the Central European winter wheat elite cultivars

Despite strong QTL × *E* interactions, 15% of the QTL were identified in two or more environments with five QTL being in common to more than one Exp. The four MEQ for which shared MTAs across Exp had been identified (Q1A_3.92_10.73, Q2A_0.47_39.79, Q2B_112.61_207.39 and Q6A_607.21_617.66) were of particular interest since these QTL promise to be more stable than loci specific for a single Exp. As detailed below, this notion is substantiated as all four loci had been identified in field trials as major *Yr* loci for Central European environments, a hot spot for the occurrence of virulent *Pst* races (Hovmøller et al. 2016).

MEQ Q2A_0.47_39.79 was detected in all three Exp and showed significant associations with dominance effects in six environments. It colocalized with an introgression harboring *Yr17* which is present in many elite cultivars (Schulthess et al. 2022). This introgression was derived from *Aegilops ventricosa* and recently sequenced (Liu et al. 2025). The substantial variation in YR severity reported here for the genomic region around *Yr17* aligns with studies on Central European elite germplasm (Miedaner et al. 2020, 2024; Beukert et al. 2020; Rollar et al. 2021). The dominant behavior of the MEQ locus Q2A_0.47_39.79 points to a race-specific disease resistance gene which are often encoded by *NLR* genes (Jones et al. 2024). Six out of seven *NLR* genes mapping to the QTL region showed nonsynonymous substitutions and thus represent candidate genes. As the resistance gene was introgressed from *Aegilops ventricosa* into the bread wheat genome, the resistance gene may represent such a diverse haplotype that SNP calling based on the Chinese Spring wheat reference genome as used here may compromise the candidate gene search. In line with this, 142 out of 533 k-mers significantly associated with YR resistance in a panel of more than 4,500 genebank samples supplemented with 67 elite cultivars were not present in wheat pangenome assemblies (Schulthess et al. 2022). Two of these mapped close to a candidate homologous to a rice blast resistance gene which had been identified in the *Aegilops ventricosa* genome segment corresponding to the introgression (Liu et al. 2025).

MEQ Q2B_112.61_207.39 was detected in Exp II and Exp III in two environments each. This region encompassed *TraesCS2B03G0442800* which corresponds to the cloned gene *Yr27* (Athiyannan et al. 2022), an allele of *Lr13* (Hewitt et al. 2021; Yan et al. 2021). Sixteen of the significantly associated SNPs in the QTL region represented nonsynonymous substitutions, with 12 mapping to *TraesCS2B03G0442800*. That this locus plays a role for YR resistance in Central European winter wheat cultivars has been suggested previously (Beukert et al. 2020; Rollar et al. 2021; Shahinnia et al. 2022; Miedaner et al. 2024).

QTL Q6A_607.21_617.66 was identified in the same environments as Q2B_112.61_207.39 and like *Yr27*, is present in the Central European breeding pools (Beukert et al. 2020; Rollar et al. 2021; Kale et al. 2022; Schulthess et al. 2022; Shahinnia et al. 2022; Miedaner et al. 2024). Remarkably, it colocalized with the most stable QTL which was observed for a European spring wheat panel that had been assessed in locations on four different continents, pointing to an *APR* locus (Lin et al. 2023). In contrast to *Yr27*, a QTL in the same region as Q6A_607.21_617.66 was also detected in plant genetic resources (Schulthess et al. 2022). Notably, the locus was more frequent in more recently released cultivars, both in spring (Lin et al. 2023) and winter wheat (Kale et al. 2022). Two genes harboring LRR domains were nominated as candidates for this MEQ.

MEQ Q1A_3.92_10.73 maps to a similar region as a QTL classified as an *APR* locus which has been identified in the winter wheat cultivar *USG 3555* (Christopher et al. 2013) and a locus in the cultivar *Madsen* which showed partial resistance at both seedling and adult plant stages (Liu et al. 2018). The observations for the latter locus concur with findings which were made for a QTL on the short arm of chromosome 1A, suggesting identity of these QTL (Rollar et al. 2021). Two MTAs representing nonsynonymous SNPs mapped to the *NLR* gene *TraesCS1A03G0032200*.

### Pyramiding genes through hybrid breeding

Single race-specific resistance genes are often overcome by evolving pathogen races. The introduction of multiple race-specific resistance genes into a single cultivar—also known as gene stacking—has therefore been advocated to achieve more durable resistance. It has been proposed that both race-specific and quantitative resistance genes need to be considered for efficient gene pyramiding (Mundt 2018; Hafeez et al. 2021). However, stacking multiple genes through conventional breeding requires many generations, particularly when integrating them into elite germplasm. In contrast, hybrid breeding offers a more efficient alternative: If a resistance locus is dominant or partially dominant, it may be sufficient to fix it in only one parental pool to achieve resistance in the hybrid offspring. Our analysis of the degree of dominance for the four major-effect QTL which had been detected across more than one Exp revealed partial dominance for most environments (Supplemental Table S4). The deviations from additivity were such that the heterozygous class generally showed resistance levels closer to the more resistant homozygous class. This indicates that the presence of resistance alleles in both heterotic pools would be advantageous. Nevertheless, combining complementary, partially dominant resistance loci at the heterozygous state is also a viable and practical approach to gene stacking, especially when considering the broad range of traits that must be integrated into elite wheat cultivars.

## Conclusion

- We generated a dataset including over 5,000 wheat hybrids and 597 lines, with more than 30,000 yellow rust (YR) scores spanning 19 environments, alongside whole-genome resequencing (WGS) data. This dataset meets the requirements for dissecting genotype-by-environment (*G* × *E*) interactions.
- We developed an association mapping approach to identify environment-specific QTL and dissect *G* × *E* interactions underlying YR resistance in Central European wheat, expanding the methodological repertoire available for genetic dissection of complex traits.
- QTL patterns varied across environments, reflecting spatial and temporal dynamics, while *G* × *E* interactions were supported by the susceptible cultivar ‘Avocet’ alongside 21 lines carrying distinct *Yr* genes over nine years.
- The dominant effects of QTL were more stable across environments than additive effects, suggesting that hybrid breeding could be a viable strategy for improving wheat resistance stability.
- A data mining strategy capitalizing on high-resolution SNP information, sequence variant annotation and QTL information from three different populations was developed for candidate gene nomination. The candidate gene approach was supported by overlap with the cloned resistance gene *Yr27* and functional gene annotations.

## Supplementary Information

Below is the link to the electronic supplementary material.

Supplementary file1 (DOCX 1269 KB)

Supplementary file1 (XLSX 2637 KB)

## Data Availability

The raw sequence data generated in this study have been deposited at the European Nucleotide Archive (PRJEB48738, https://www.ebi.ac.uk/ena/browser/view/PRJEB48738). Parental lines from Exp I were previously released and described in Schulthess et al. (2022) and parental lines from Exp II and III (PRJEB82869, https://www.ebi.ac.uk/ena/browser/view/PRJEB82869) were released and described in Li et al. (2025). The VCF files corresponding to WGS data have been deposited in the European Variation Archive (EVA) under Project (PRJEB87554, https://www.ebi.ac.uk/eva/?eva-study=PRJEB87554). The phenotypic data (BLUEs) across environments for all parental lines and hybrids from three experiments can be found in Supplemental Table S9 of this article.
