# Supplementary material for: From QTL to candidate genes: a data-driven approach to unravel the genetic architecture of yellow rust resistance in central European wheat
Source: Theor Appl Genet. 2026 Jul 30;139(8):219. doi: 10.1007/s00122-026-05312-8 (PMC13423923; doi:10.1007/s00122-026-05312-8)
Supplement: Supplementary file 1 — Supplementary file1 (DOCX 1269 KB) [file 122_2026_5312_MOESM1_ESM.docx]

**Supporting Information**

**Supplemental Figures**


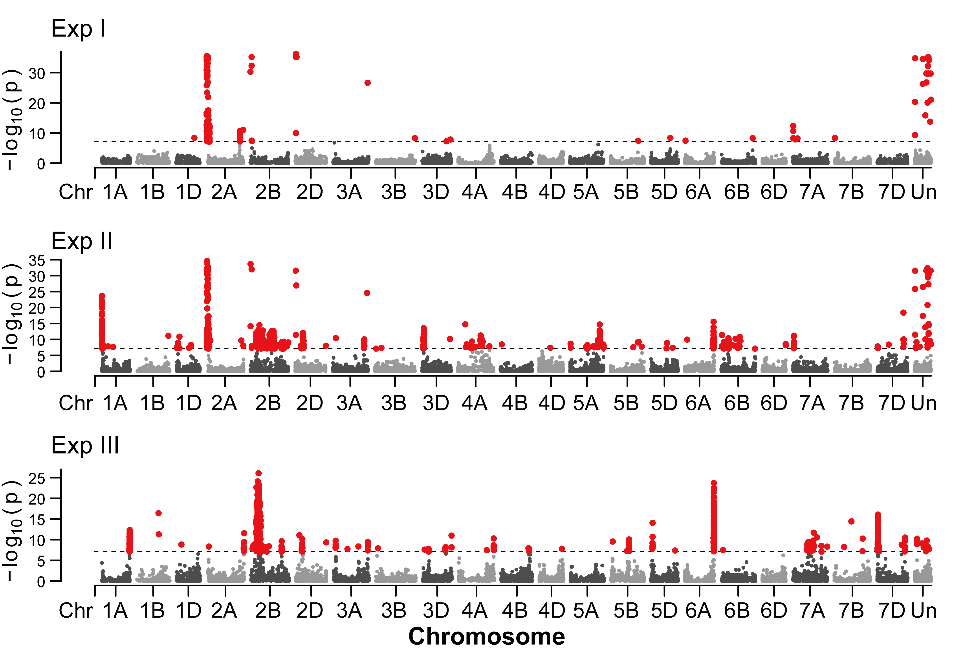


**Supplemental Figure 1. The marker-trait association for G×E interaction effects from the joint test.**

Manhattan plots showing the results of GWAS for interaction effects between markers and environments using the joint test of additive and dominance effects. In each experimental series (Exp), the genome-wide threshold of *p* < 0.05 after Bonferroni correction was indicated by the dashed horizontal line.


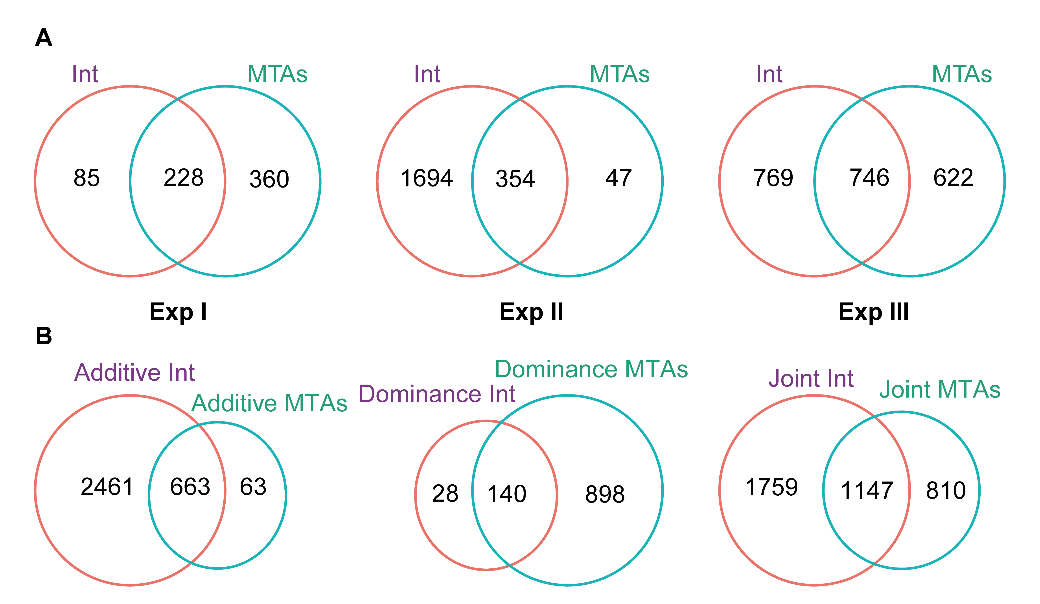


**Supplemental Figure 2. Overlap between interaction effects between markers and environments (Int) and marker-trait associations (MTAs) across experimental series (Exp).**

**(A)** Venn diagrams displaying the number of significant Int effects and MTAs (combining results of additive, dominance, and joint test) identified in the three independent experimental series (Exp I, Exp II, and Exp III). **(B)** Overlaps between Int and MTAs are shown separately for the three genetic effect categories—additive, dominance, and joint test. The results of all Exp were combined. The numbers within each circle add up to the count of significant markers identified in each category, while the overlapping regions between circles represent shared markers.


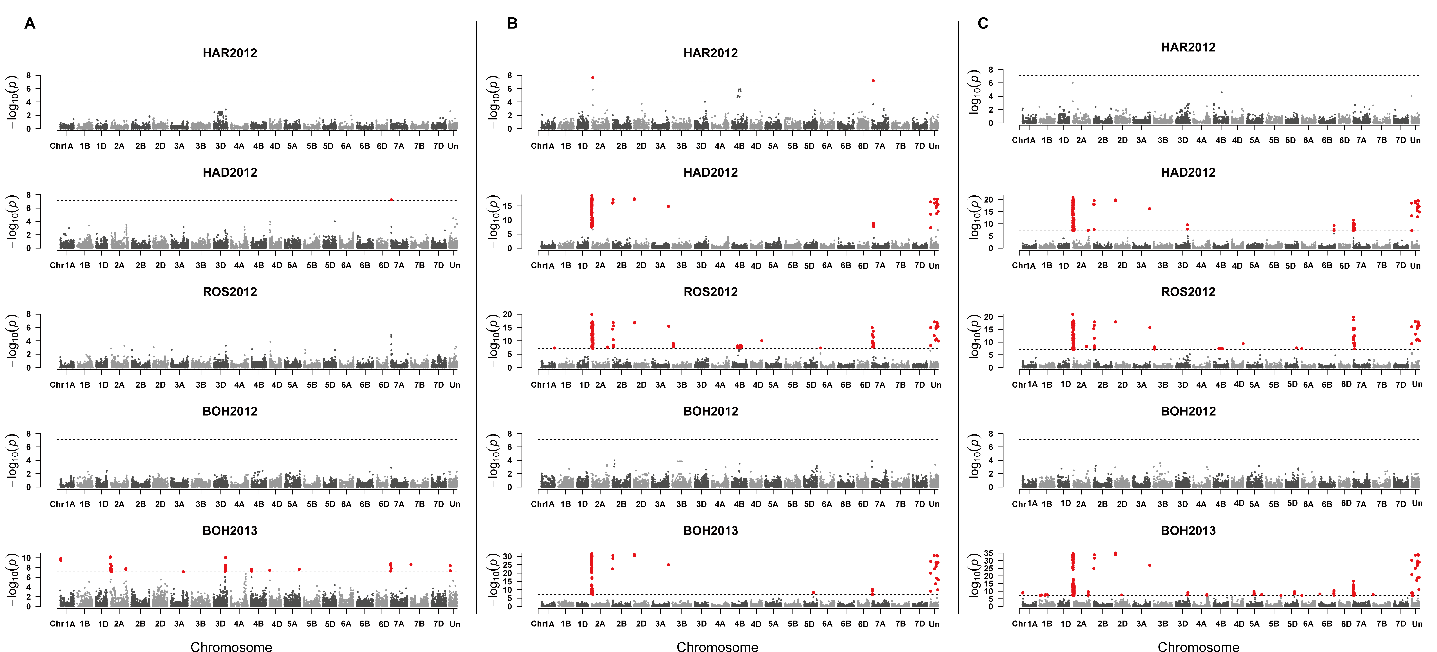


**Supplemental Figure 3. Manhattan plots showing the results of GWAS within particular environments of experimental series I (Exp I) under different tests.**

The different panels refer to the additive **(A)**, dominance **(B)** and joint test **(C)**. The genome-wide threshold of *p* < 0.05 after Bonferroni correction was indicated by the dashed horizontal line in each of the plots.

**
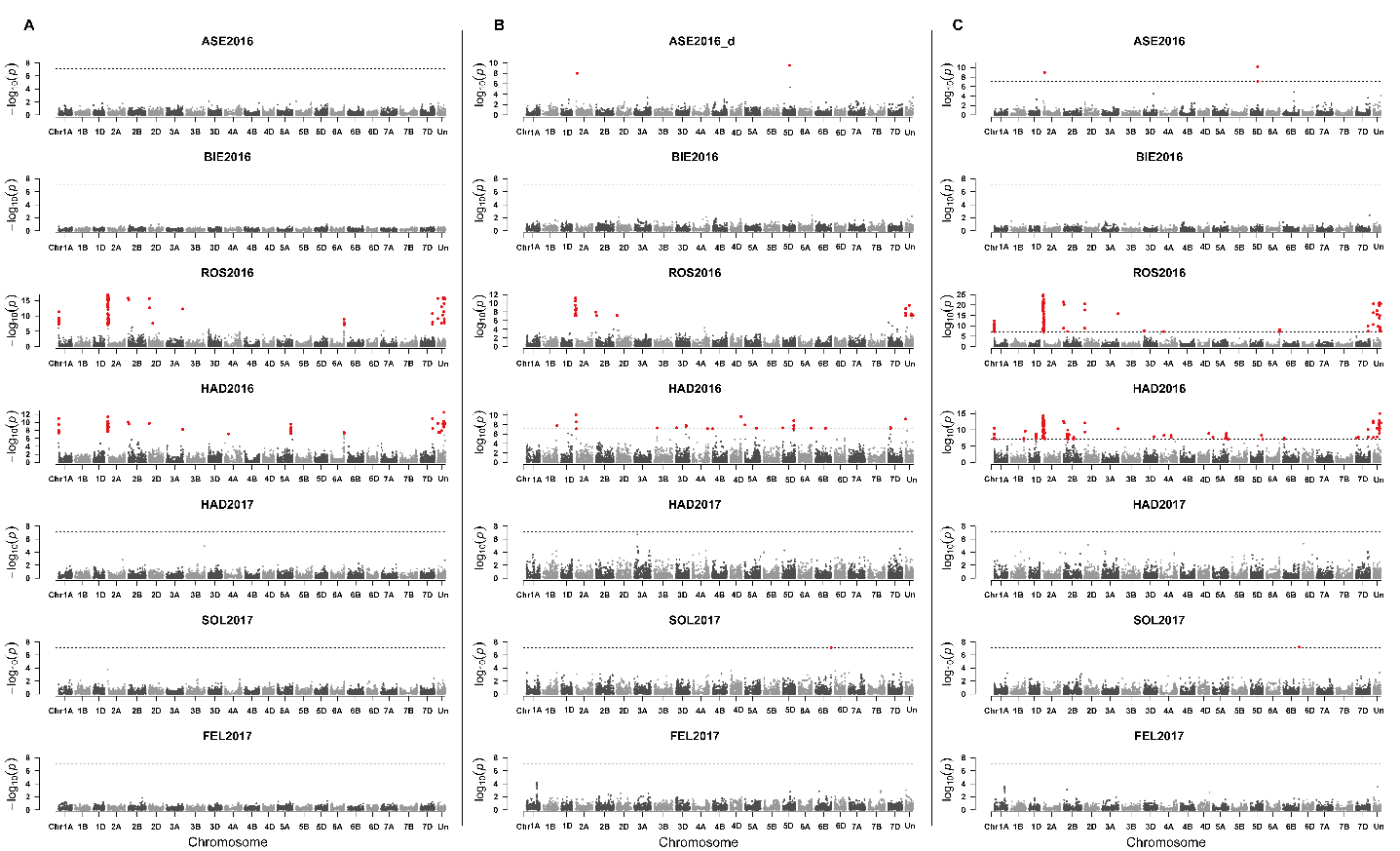
**

**Supplemental Figure 4. Manhattan plots showing the results of GWAS within particular environments of experimental series II (Exp II) under different tests.**

The different panels refer to the additive **(A)**, dominance **(B)** and joint test **(C)**. The genome-wide threshold of *p* < 0.05 after Bonferroni correction was indicated by the dashed horizontal line in each of the plots.

**
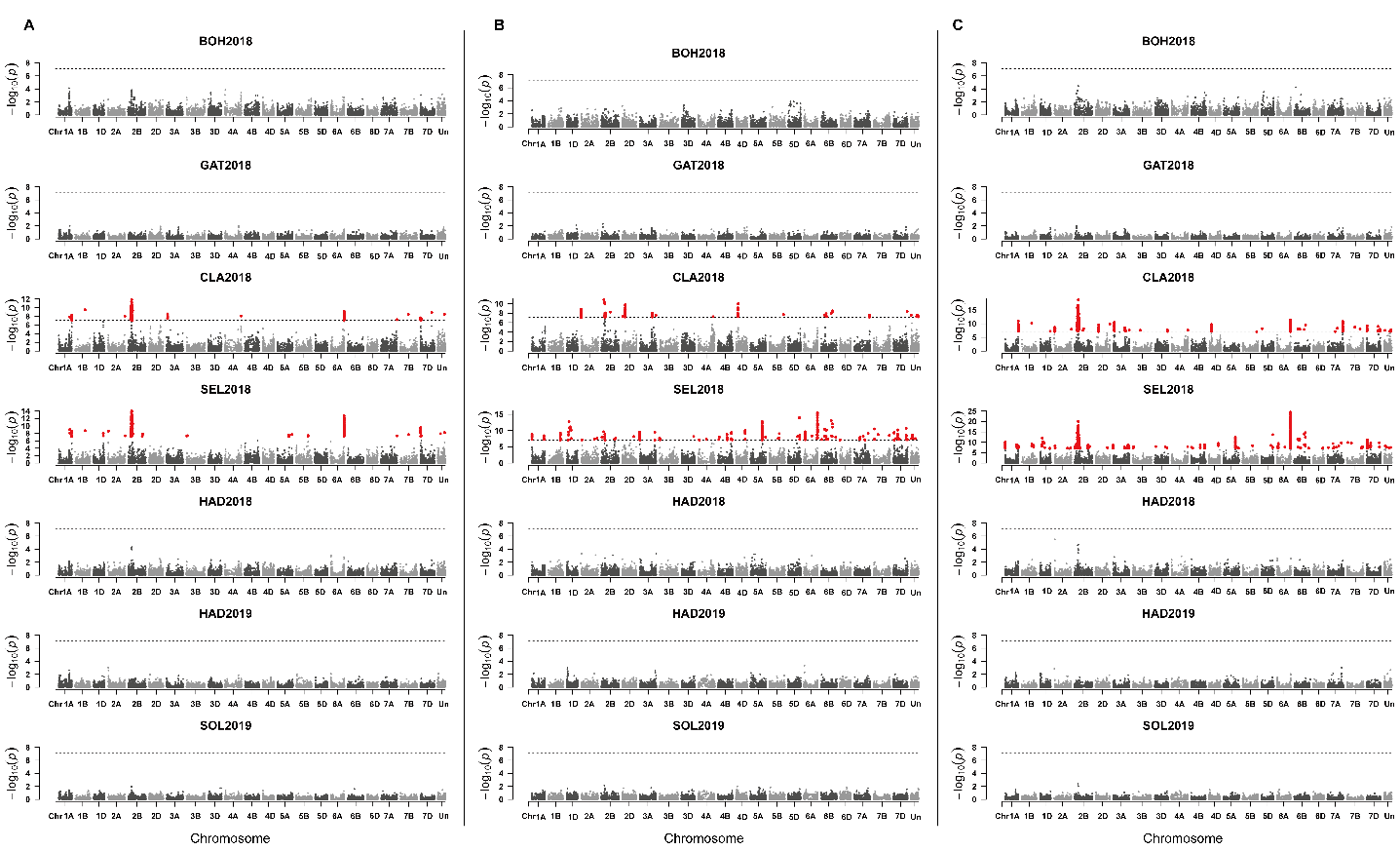
**

**Supplemental Figure 5. Manhattan plots showing the results of GWAS within particular environments of experimental series III (Exp III) under different tests.**

The different panels refer to the additive **(A)**, dominance **(B)** and joint test **(C)**. The genome-wide threshold of *p* < 0.05 after Bonferroni correction was indicated by the dashed horizontal line in each of the plots.


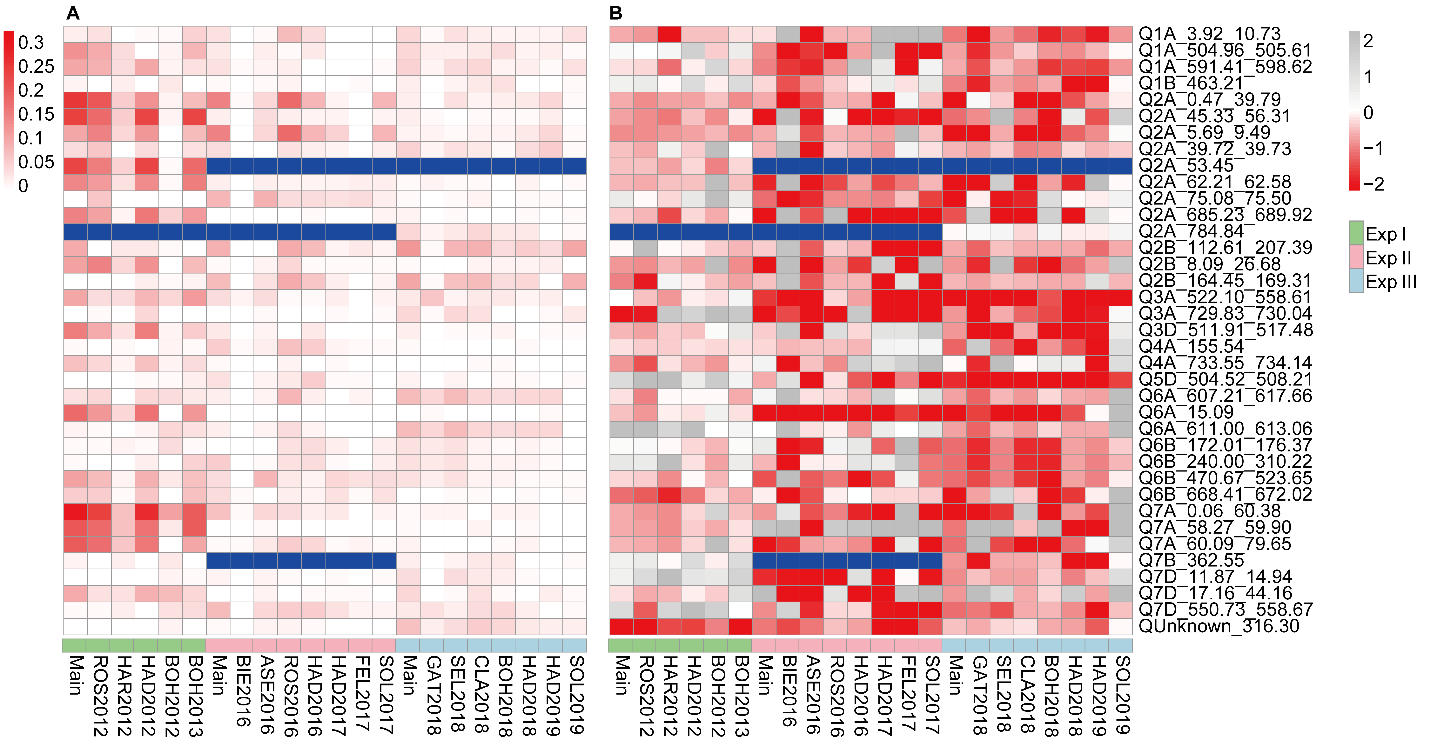


**Supplemental Figure 6. PVE and dominance effects of MEQ across three experimental series.**

The PVE **(A)** and degree of dominance (**B**) of 37 MEQ detected by the joint test of additive and dominance effects within each environment in three experimental series (Exp). Negative degrees of dominance indicate resistance in this study. In all cases, the estimates were based on the lead SNP representing the QTL. Blue: missing values, indicating that the relevant SNPs could not be analyzed in this particular Exp.

**Supplemental Tables**

**Supplemental Table 1. Summary of wheat populations evaluated in field trials across three different experimental series (Exp) and the number of single nucleotide polymorphisms (SNPs) identified through whole genome sequencing (WGS)**

| Experimental series (Exp) | No. of environments | No. of checks | No. of females | No. of males | No. of hybrids | No. of genotypes | No. of SNPs |
| --- | --- | --- | --- | --- | --- | --- | --- |
| I | 5 | 10 | 120 | 15 | 1,604 | 1,749 | 655,604 |
| II | 7 | 11 | 185 | 41 | 1,815 | 2,052 | 646,722 |
| III | 7 | 11 | 196 | 40 | 1,824 | 2,071 | 622,069 |

**Supplemental Table 2. Site information used for the hybrid yield trials**

| Experimental series (Exp) | Location | Year | Environment | Latitude | Longitude | Altitude |
| --- | --- | --- | --- | --- | --- | --- |
| I | Böhnshausen | 2012 | BOH2012 | 51°51'30.953" N | 10°57'44.32" E | 173 m |
| I | Böhnshausen | 2013 | BOH2013 | 51°51′ N | 10°57′ E | 146 m |
| I | Hadmersleben | 2012 | HAD2012 | 51°59'29.785" N | 11°18'12.794" E | 91 m |
| I | Harzhof | 2012 | HAR2012 | 54°24′N | 9°51′ E | 25m |
| I | Rosenthal | 2012 | ROS2012 | 52°18' N | 10°10' E | - |
| II | Biendorf | 2016 | BIE2016 | 51°45'0'' N | 11°50'59'' E | 79 m |
| II | Asendorf | 2016 | ASE2016 | 52°44'17.934'' N | 9°0'24.105'' E | 45 m |
| II | Rosenthal | 2016 | ROS2016 | 52°18'18.89'' N | 10°10'52.88'' E | 70 m |
| II | Feldkirchen | 2017 | FEL2017 | 48°29′21.7'' N | 48°29′21.7'' E | - |
| II | Söllingen | 2017 | SOL2017 | 52°05′48.9''N | 10°55′34.0'' E | - |
| II | Hadmersleben | 2016 | HAD2016 | 51°59'29.785" N | 11°18'12.794" E | 91 m |
| II | Hadmersleben | 2017 | HAD2017 | 51°59'29.785" N | 11°18'12.794" E | 91 m |
| III | Gatersleben | 2018 | GAT2018 | 51°82′41.77'' N | 11°27′57.06'' E | 112 m |
| III | Seligenstadt | 2018 | SEL2018 | 49°51'16.30'' N | 10°06'2.30'' E | 280 m |
| III | Clauen | 2018 | CLA2018 | 52°259′ N | 10°031′ E | 84 m |
| III | Böhnshausen | 2018 | BOH2018 | 51°51′ N | 10°57′ E | - |
| III | Söllingen | 2019 | SOL2019 | - | - | - |
| III | Hadmersleben | 2018 | HAD2018 | 51°59'29.785'' N | 11°18'12.794" E | 91 m |
| III | Hadmersleben | 2019 | HAD2019 | - | - | - |

**Supplemental Methods**

**Additional field trials**

The Australian spring wheat cultivar *Avocet*, which is highly susceptible to YR, and 21 genotypes carrying different resistance genes were evaluated. There are three genotypes each containing two *Yr* genes (*Yr22+Yr23*, *Yr29+Yr31, Yr73+Yr74*), and 18 genotypes each carrying one of the following genes: *Yr1*, *Yr5*, *Yr6*, *Yr7*, *Yr8*, *Yr9*, *Yr10*, *Yr15*, *Yr17*, *Yr18*, *Yr19, Yr24*, *Yr26*, *Yr27*, *Yr28*, *Yr32* (*YrCV*), *Yr35* and *YrSP*. Field trials were conducted annually from 2015 to 2024 at the field station in Quedlinburg, Germany (51°46′22.22″ N, 11°9′12.82″ E; 140 m a.s.l.; clayey loam black soil; mean annual temperature 8.9 °C; mean annual precipitation 497 mm). Genotypes were sown in two rows 15 cm apart with a length of one meter in a completely randomized design with three replicates. Artificial inoculation was performed using a mixture of *Puccinia striiformis* f. sp. *tritici* (*Pst*) isolates collected in previous seasons. Spreader rows of the susceptible cultivar *Triso* were sown in every third plot to promote uniform disease development. Phenotyping was conducted by visually assessing the percentage of infected leaf area on the second and third youngest leaves of two rows per plot, across four consecutive rating dates. Assessments began when infection symptoms were clearly visible on the spreader plots, defined as ≥10% infected area on *Triso* leaves, and were repeated every 7–9 days. For each genotype and year, the infection level was calculated as the area under the disease progress curve (AUDPC, Moll et al., 1996), based on the percentage of infected leaf area at each time point.

**Supplemental References**

Moll, E., Walther, U., Flath, K., Prochnow, J., and Sachs, E. (1996) *Methodische Anleitung zur Bewertung der partiellen Resistenz und die SAS-Anwendung RESI*. Eigenverlag: Braunschweig, Germany. doi:10.5073/20210115‑120959
